# Analgesia Synergism of Essential Oil from Pericarp of *Zanthoxylum schinifolium* and Verapamil

**DOI:** 10.1155/2014/505876

**Published:** 2014-07-08

**Authors:** Gao Wu, Hanbin Wu

**Affiliations:** ^1^Department of Pharmacy, The 411st Hospital of PLA, Shanghai 200081, China; ^2^Clinical Pharmacy, Shanghai East Hospital, Tongji University School of Medicine, Shanghai 200120, China

## Abstract

*Objective*. To evaluate the synergistic analgesic effect of essential oil of *Zanthoxylum schinifolium* Sieb. et Zucc. (EOZ) and verapamil (Ver). *Method*. The qualitative and quantitative composition of EOZ were determined with gas chromatography/Mass spectrometer. The interaction between EOZ and Ver in antinociceptive activity was evaluated by using acetic acid-induced writhing, hot plate, and tail flick tests in mice and in isolated toad sciatic nerve test. *Results*. Linalool, limonene, and sabinene are the major components of EOZ. EOZ (middle-dose: 40 mg*·*kg^−1^, high-dose: 80 mg*·*kg^−1^) and EOZ + Ver (Each dose group) have remarkable analgesic effects on pain in mice induced by acetic acid-induced writhing, hot plate, and tail flick tests. Low-dose EOZ (20 mg*·*kg^−1^) had no analgesic action, but when it is combined with Ver it has shown significant antinociception. Verapamil has a faint analgesic effect but was not able to inhibit action potential transmission in toad sciatic nerve. EOZ (0.2%) and EOZ + Ver (0.2% + 0.05%) also inhibited action potential transmission in toad sciatic nerve. Combination of EOZ with Ver had a greater analgesic effect and inhibition of nerve action potential transmission compared to its components EOZ and Ver. *Conclusion*. The combination of EOZ with Ver produces a synergistic analgesic effect.

## 1. Introduction

There are many different names for the* Zanthoxylum* genus in China, with the most popular name being “huajiao” (flower pepper), which refers to the group of related species. The two most commercially popular species are* bungeanum* (red huajiao) and* schinifolium* (green huajiao). The pericarps of several* Zanthoxylum* species are used in China and other East Asian countries as a kind of spice and in traditional Chinese medicine for their therapeutic properties [1]. It is effective for the treatment of inflammatory diseases, epigastric pain, stomachache, toothache, ascariosis, diarrhea, and dysentery. In addition, the pericarps are also used as antimicrobials, insect repellents, antioxidants, and feeding deterrents. These functions are due to the pericarp essential oil [2, 3]. It has antioxidant, blood lipid regulating, antiplatelet, antithrombotic, and antihypertensive properties, which provide protection from stress-induced myocardial injury, as well as anti-inflammatory, analgesic, immunomodulatory, and antitumor functions [4]. Those effects are attributed to the monoterpenes, which are the major chemical components of the essential oils. Limonene and linalool are monoterpene prevalent in the essential oils. Fresh huajiao has a very high content of essential oil, up to 11%. A total of 120 aroma compounds for each species have been found. In the essential oils, linalyl acetate (15%), linalool (13%), and limonene (12%) are the major components of red huajiao, whereas linalool (29%), limonene (14%), and sabinene (13%) are the major components of green huajiao [5–8]. As previously reported linalool and limonene produce antinociceptive activities in several behavioral assays. Green huajiao presented a higher antinociceptive effect than red huajiao, which has higher linalool and limonene content compared to red huajiao [9–11].

Pain is an unpleasant sensory and emotional experience that is associated with actual or potential tissue damage. Calcium plays an important role in the transmission of pain signals in the central nervous system [12]. At the presynaptic nerve terminal, voltage-gated calcium channels (VGCCs) open in response to action potentials to allow an influx of calcium ions. The influx, in turn, leads to the release of various neurotransmitters that diffuse across the synaptic cleft to the postsynaptic membrane and bind to their specific receptors. The binding of morphine to *μ*-opioid receptors leads to the inhibition of neurons concerned with the transmission of pain. The *μ*-opioid receptor does so by blocking VGCCs, opening inwardly rectifying potassium channels and inhibiting the activity of adenylyl cyclase [13]. The release of pain-producing neurotransmitters like substance P from the presynaptic terminals in the spinal cord is thereby decreased, leading to relief from pain [14]. A number of studies have shown an increase in the analgesic response to opioids like morphine, when coadministered with L-type calcium channel blockers (CCBs) [15–18].

Experiments show that the essential oil of* Zanthoxylum* (EOZ) inhibits the contraction of uterine smooth of rat and colon smooth of rabbit, mainly by blocking calcium channels and consequently decreasing the influx of extracellular calcium and release of intracellular calcium [19, 20]. Therefore, the present study was undertaken to examine whether EOZ and the calcium channel blocker verapamil have a synergistic analgesic effect.

## 2. Materials and Methods

### 2.1. Plant Material and Reference Drugs

The pericarps of* Zanthoxylum* (green huajiao) were purchased at Shanghai Hongqiao Chinese Herbal Medicine Co., Ltd. The plant material was identified as the pericarp of* Zanthoxylum schinifolium* Sieb. et Zucc.: 1% lidocaine (Huarui Pharmacy, Wuxi, Jiangsu, China), Aspirin (Shanghai Pharmaceutical Co., Ltd. Xinyi Pharmaceutical Factory, Shanghai, China), verapamil (Sigma Pharmaceutical Industrial Co.), and morphine hydrochloride injection (Shenyang first pharmaceutical factory, Shenyang, China).

### 2.2. Animals

Kunming mice (25–30 g) were purchased from Shanghai Experimental Animal Center (Shanghai, China) and raised in our specific pathogen-free and air-conditioned animal facility. All of the experiments were performed with the sciatic nerves dissected from wild adult toads (Bufo bufo gargarizans Cantor) weighing 60–90 g.

### 2.3. Preparation and Analysis of Essential Oil of* Zanthoxylum bungeanum*

Essential oil of* Zanthoxylum schinifolium* (EOZ) was extracted using a modified Clevenger apparatus by the hydrodistillation technique. The obtained EOZ was dried over sodium sulfate and used as the basic material. EOZ was stored in hermetically sealed glass receptacles with rubber stoppers, covered with aluminum foil to protect the contents from light, and kept under refrigeration at 4°C until use.

The components of the EOZ were analyzed using GC/MS-QP 2010 (Tokyo, Japan), with an autoinjector (AOC-20i) and autosampler (AOC-20s). Sample was eluted with Helium gas. Components were separated with capillary column (Rtx-1 MS Prepared by Restek Corporation USA) having 30 m × 0.250 mm and 0.25 micrometer thickness. Electronimpact ionization mode was with energy 70 eV, ion source temperature 200°C, interface temperature 250°C with 28.8 KPa pressure, and 1.8 min solvent cut time. Injector temperature was 250°C and operated in split mode with 2 mL/min. The column was programmed at a temperature of 40°C for 3 min initially and then changed to 150°C at the rate of 15°C/min and kept constant for 15 min. The column temperature was increased to 250°C at a rate of 5°C per min and was maintained for 3 min. Mass spectra were acquired in the range of 20 to 400 m/z. A series of normal alkanes was also injected under same analytical conditions with that of the essential oil for the calculation of retention indices. Components of the essential oil were identified by comparing the mass spectra obtained with those of standard mass spectra from the NIST library (NIST 08). Relative concentration of the components was calculated from the peak areas of the total ion chromatograms.

### 2.4. Evaluation of Antinociceptive Activity of EOZ

#### 2.4.1. Acetic Acid-Induced Writhing Test

The method previously described was used to evaluate the antinociceptive activity [21]. Kunming mice, of either sex, were injected with 0.6% acetic acid in 0.9% normal saline (0.2 mL) by intraperitoneal injection. The number of writhings within 15 min was then recorded, and the writhing number was regarded as the pain threshold. The selection of qualified mice involved choosing those in which the writhing number within 15 min was 11~59, those in which the writhing number was more than 60 times or less than 10 times were abandoned. Mouse button on the standard body followed by the writhing number, and then per ten mice with similar writhing number as a group, were randomly assigned to nine different experimental groups: the control group (0.9% normal saline, NS, 60 mg·kg^−1^, p.o.), EOZ different groups (at the doses of 20 mg*·*kg^−1^, 40 mg*·*kg^−1^, and 80 mg*·*kg^−1^, p.o.), Ver group (5 mg*·*kg^−1^, p.o.), combined EOZ + Ver different groups (at the doses of 20 mg*·*kg^−1^ + 5mg*·*kg^−1^, 40 mg*·*kg^−1^ + 5 mg*·*kg^−1^, and 80 mg*·*kg^−1^ + 5 mg*·*kg^−1^, p.o.), and reference Asp group (aspirin, 200 mg·kg^−1^, p.o.). A week later, experimentation began, and all test drugs were given orally to the separate groups of mice prior to acetic acid injection. The mice were observed and counted for the number of abdominal constrictions and stretchings in a period of 0–20 min. The responses of the mice in the treated groups were compared with those animals in the control group. The percentage of inhibition of the number of writhings was calculated.

#### 2.4.2. Hot Plate Test

Kunming female mice were used [22]. The hot plate was an electrically heated iron surface of water bath kept at a constant temperature of 55.0 ± 0.5°C After 30 min of treatment (except only 15 min for morphine) with all test drugs, the control group (0.9% normal saline, NS, 60 mg·kg^−1^, p.o.), EOZ different groups (at the doses of 20 mg·kg^−1^, 40 mg·kg^−1^, and 80 mg·kg^−1^, p.o.), Ver group (5 mg·kg^−1^, p.o.), combined EOZ + Ver different groups (at the doses of 20 mg·kg^−1^ + 5mg·kg^−1^, 40 mg·kg^−1^ + 5 mg·kg^−1^, and 80 mg·kg^−1^ + 5 mg·kg^−1^, p.o.), and reference Mor group (morphine, 10 mg·kg^−1^, i.p.) mice (*n* = 10 per group) were placed on the heated surface, with Plexiglas walls to constrain their locomotion on the plate. The latency to a discomfort reaction (licking of the paws or jumping) was recorded at 15, 30, 60, 90, and 120 min after drug treatment; the reaction time of 0 min was the start of the test. A cut-off time of 60 s was chosen to indicate complete analgesia and to avoid tissue injury. Only mice that showed a nociceptive response within 15 s were used in the experiments.

#### 2.4.3. Tail Flick Test

This test was applied as described above [21]. The lower two-thirds of the tail was immersed in a beaker containing hot water kept at 50 ± 0.5°C. The time in seconds until the tail was withdrawn from the water was defined as the reaction time. The reaction time was then measured 0, 15, 30, 60, 90, and 120 min after 30 min of treatment (except only 15 min for morphine) with all test drugs (NS, EOZ, Ver, EOZ + Ver, and Mor); the reaction time of 0 min was the start of the test. Mice (*n* = 10 per group) showing a pretreatment reaction time greater than 5 s in the tail flick test were not used in the experiment. A cut-off time of 20 s was set to avoid tissue damage.

#### 2.4.4. Effect on Isolated Toad Sciatic Nerve

The toads were rapidly decapitated and killed [23], exposed both sides of the sciatic nerve lumbar plexus nerves to the legs of blunt isolated and immediately maintained in Ringer solution (RS). Fifty isolated toad sciatic nerves were selected and randomly assigned to 5 groups of 10 each: control group (RS), EOZ + Verapamil compound group (EOZ + Ver, 0.2% EOZ + Ver 0.05%), EOZ group (EOZ, 0.2%), verapamil group (Ver, 0.05%), and lidocaine group (Lid, 1% lidocaine). Compound nerve action potential (CNAP) was recorded via an extracellular recording technique with a BL-420F Acquisition System (Taimeng Technology, Chengdu, China). After 30 minutes of stabilization in RS, segments of nerve measuring 5 cm were placed in a Plexiglas nerve chamber. The space between the electrodes was fixed during the entire procedure. The stimulating voltage was set to produce a maximal CNAP using single square pulses of supra maximal strength and 0.5 milliseconds in duration. Then put the drug's cotton balls (cotton balls as rare as possible, the same size) on the nerves (between the stimulation side and recording-side), and the added liquid is about 0.05 mL. At 1, 5, and 10 min after dosing, the waveform changes and action potential wave disappearance time were observed and recorded.

### 2.5. Statistical Analysis

The same subject was observed using repeated-measure design. In this design, each subject serves as its own control. Results are presented as mean ± SEM, with (*N*) indicating the number of subjects. All analyses were performed using the SPSS 13.0 statistical software (SPSS, Chicago, IL). Statistically significant differences between groups were calculated by the application of analysis of variance (ANOVA) followed by Bonferroni's test. The independent* t*-test was used for comparison between 2 groups. *P* values less than 0.05 (*P* < 0.05) were used as the significance level.

## 3. Results

### 3.1. Analysis of Essential Oil of* Zanthoxylum bungeanum* (EOZ)

The percentage yield of essential oil based on the dried pericarp was 2.82% (v : w). Composition of essential oil has been summarized in Table 1. Linalool, d-limonene, and sabinene are the major constituents of the essential oil.

### 3.2. Acetic Acid-Induced Writhing Test

All oral administrations of test drugs except EOZ low-dose group caused a significant reduction in the number of writhing episodes induced by acetic acid compared to the control (*P* < 0.05 and *P* < 0.01). The combined EOZ + Ver groups resulted in 52.8%, 34.9, and 13.4% reduction of writhing episodes number compared to the relevant sole EOZ groups, respectively. It showed that low-dose EOZ group had no significant difference with control group in the number of writhing episodes (>0.05), but it is combined with Ver; writhing number could be reduced by 52.8% and was similar to the reference group (aspirin group), which is more obvious than that of the middle and high dose of EOZ (34.9 and 13.4% resp.). The analgesic effect was weaker in the sole EOZ group whose combined EOZ + Ver group resulted in an increase greater degree of synergistic analgesic effect. The results are provided in Table 2.

### 3.3. Hot Plate Test

There was no significant difference between each treatment group's reaction time at 15 min after administration and the reaction time before administration, but there was an increasing trend, except in the reference Mor group. All drug groups (EOZ + Ver, EOZ) except Ver group and low-dose EOZ group considerably increased the animal's reaction time to the heat stimulus after 30 min. This indicates that their analgesic effects of EOZ come into play at about 30 min or so and are maintained for at least 120 min; however, Mor was only maintained for 60 min. Although the Ver and low-dose EOZ produced no significant increase in the reaction time throughout the observation period, the analgesic effect of low-dose combined EOZ + Ver was significantly (*P* < 0.01) greater than that of the relevant low-dose EOZ after 30 min. The analgesic effect of all EOZ + Ver groups were significantly (*P* < 0.01) greater than that of the relevant sole EOZ groups, respectively, indicating that EOZ and Ver have a synergistic analgesic effect for the hot plate test. The results are provided in Table 3.

### 3.4. Tail Flick Test

After treatment administration, there was no significant difference in the tail flick latency (TFL) between each treatment group's reaction time at 15 min, except in the reference Mor group. All treatment groups except the Ver group and low-dose EOZ group significantly (*P* < 0.001 and *P* < 0.05, resp.) increased the tail flick latency in 30 min to 120 min observation period, as compared with the control group. All combined EOZ + Ver groups showed a significant (*P* < 0.05) increase in the reaction time when compared with the relevant sole EOZ group, respectively. This indicates that EOZ and Ver have a synergistic analgesic effect for the tail flick test. The results are provided in Table 4.

### 3.5. Isolated Toad Sciatic Nerve Test

No significant differences were found in the baseline values of nerve action potential amplitude among the 5 groups. The negative amplitude after drug administration continuously declined in the combined EOZ + Ver group and the EOZ group, as well as in the Lid group. In contrast, the negative amplitude in the RS group and Ver group remained stable. The conduction blockade induced by EOZ + Ver and EOZ had a faster onset (amplitude begins to decrease) and the action potential disappeared faster as compared with lidocaine (onset: 1 minute, 1 minute versus 10 minutes and disappear: 3 minutes, 5 minutes versus 22 minutes). For both groups, the strength of conduction blockade was greater than that of the Lid group (the negative amplitude decreased baseline 46% and 27% versus 3%). The disappearance of the action potential of the combined EOZ + Ver group was faster than those of the EOZ group, suggesting that the combination of EOZ and Ver had a synergistic effect in the isolated toad sciatic nerve test. The results are provided in Table 5.

## 4. Discussion

The percentage yield of essential oil based on the dried pericarp was 2.82% (v : w). Linalool, d-limonene, and sabinene are the major constituents of the essential oil. The results are consistent with those reported [5].

In the present study, our results demonstrated that Ver showed an analgesic effect only in writhing reaction; in the hot plate test and tail-flick test, Ver alone did not produce an analgesic effect. However, when verapamil was combined with EOZ, its analgesic effect is obviously enhanced as shown by the three animal experiment results. Low-dose EOZ had no analgesic action, but when it is combined with Ver it has shown significant antinociception which was similar to middle and high dose combined EOZ + Ver. It indicated that Ver could not only enhance the analgesic action of EOZ, but also reduce its dosage. In other words, the analgesic potency of combined EOZ + Ver groups significantly improved compared to the relevant sole EOZ groups, respectively, in the three animal experiments. Results showed that the EOZ and Ver analgesics have a synergistic effect. Calcium is a coupling factor necessary in presynaptic membrane excitability and neurotransmitter release; the release of neurotransmitters induced by noxious stimuli is related to electrical activity of VGCCs on the membrane of synaptic endplate. Experiments with VGCC antagonists revealed that L-, N-, and P/Q-, but not T-type channels, are involved in nociception, and potentiation of opioid-induced antinociception was more frequently seen with L-type antagonists [24]. The L-type CCB verapamil potentiates morphine analgesia through a peripheral mechanism. Earlier studies have shown that intrathecal administration of Ver did not show any antinociception; however, when intrathecally administered, Ver combined with ineffective or moderately effective doses of intrathecally administered morphine produced significant antinociception. These interactions were synergistic [25]. The study demonstrated that intrathecally administered L-type calcium channel blockers diltiazem or Ver produced both somatic and visceral antinociception and motor block dose dependently. Further, Ver evoked antinociception in the mouse hot-plate test, and further experiments showed that these effects might be due to the agonistic activity of verapamil at *μ*-, *δ*-, and *κ* 3-receptor subtypes. Interestingly, some of the CCBs (diltiazem and Ver) also increase morphine levels in the brain when coadministered together, as compared to morphine alone, after systemic administration [26]. Thus, Ver can have a synergistic effect on the analgesic effect of opioids. EOZ inhibits the contraction of smooth muscle mainly by blocking calcium channels, and its calcium antagonism mechanism is not exactly the same as that of Ver [19, 20]. The results of the present study indicate that Ver increases the analgesic effect of EOZ. The mechanism may be different from the analgesia synergism of Ver and opioids, and they prevent Ca^2+^ influx in different ways, which create a synergistic analgesic effect.

The present study showed that EOZ (middle and high dose) and combined EOZ + Ver possess significant antinociceptive effects as evaluated in the acetic acid-induced writhing test, hot plate test, and tail-flick test, and they inhibit the induction of action potentials in toad sciatic nerve. Ver has a weak analgesic effect and no effect in sciatic nerve action potential block. The analgesic effects and inhibition of nerve action potential conduction of combination of EOZ and Ver are greater than both component EOZ and verapamil. The acetic acid-induced writhing model is a chemical stimulus widely used for the evaluation of peripheral antinociceptive activity. In this model, acetic acid indirectly induces the release of endogenous mediators, stimulating the peripheral nociceptors and sensitive neurons that were sensitive to the inflammatory mediators. The results of this study revealed that the analgesic potency of combined EOZ + Ver groups were significantly improved than the relevant sole EOZ groups, respectively, in acetic acid-induced writhing test, similar to the reference drug aspirin (200 mg/kg). Therefore, one possible mechanism of antinociceptive activity of EOZ could be a blockade of the effect or the release of endogenous substances. EOZ has been reported to inhibit the contractions of smooth muscle [19, 20]. The antinociceptive action of EOZ observed in this study may not be involved with the inhibition of smooth muscle contractions by EOZ. The tonic inhibition of smooth muscle contractions produced by EOZ may be responsible for the inhibition of contortions but is not related to its analgesic activity. However, the results of this test do not ascertain whether the antinociceptive effect was mediated by a central or peripheral process.

To evaluate possible participation of the central analgesic system in the antinociceptive activity of EOZ, the hot plate test and tail-flick test were employed [27]. In the tail immersion test, which consists of a thermal stimulus, an increase in the reaction time is generally considered to be an important parameter for evaluating central antinociceptive activity. The hot plate test is predominantly a spinal reflex or behavioral reaction and used to test supraspinal analgesia in compounds. Both are considered to be supraspinally integrated responses. It is, therefore, selective for centrally acting analgesic drugs like morphine. EOZ was found to have antinociceptive activity in the hot plate test and tail immersion test in middle and high dose. These tests also revealed that the antinociceptive effect of EOZ on mice remained present for at least up to 120 min after administration in middle and high dose. However, morphine, a well-known opioid agonist, produced a profound antinociceptive effect to the hot plate test in the period of 60 min. The analgesic effect of all combined EOZ + Ver groups were significantly (*P* < 0.01) greater than that of the relevant sole EOZ groups, respectively, indicating that EOZ and Ver have a synergistic analgesic effect for the hot plate test and tail immersion test. In other words, the analgesic potency of EOZ was strengthened after the addition of Ver. The antinociceptive effects of EOZ involve supraspinal as well as spinal components, as demonstrated by the use of the hot plate and tail immersion tests, respectively. Therefore, taking all these data together, we believe that the antinociceptive activity of EOZ is most likely to be mediated peripherally and centrally.

The major result of isolated toad sciatic nerve test was that EOZ reversibly inhibited compound nerve action potential of the toad sciatic nerve, and Ver has synergistic analgesic effect on EOZ. The mechanism is not only related to blocking calcium influx, but may also be related to Na^+^ channel blockers. Early study shows EOZ has a local anesthesia; analgesic effect of EOZ is due to the effect of its local anesthesia. The target of local anesthetic is usually a Na^+^ channel on the inner side of the nerve cell membrane. Local anesthetics inhibit the transmission of nerve impulses in nerve fibers by blocking Na^+^ channels, producing a local anesthetic effect, and have no relation with Ca^2+^ opioid receptors. According to the theory of neural electrophysiology, formation of nerve action potential does not directly involve Ca^2+^. The nerve fiber action potential opens a large number of Na^+^ channels on the cell membrane, leading to a Na^+^ influx. Lidocaine reduces the internal flow of Na^+^ by blocking Na^+^ channels, thus inhibiting the action potential of sciatic nerve. The local anesthetic effect of EOZ has previously been observed. Thus we presume that the analgesic effect of EOZ may be related to the blocking of Na^+^ channels on nerve cell membranes, which reduces the amplitude of the action potential due to the influx of Na^+^ and produces an anesthesia effect. The analgesia synergism of the combination of EOZ with Ver is probably caused by the blocking of nerve impulses from the different way. At the presynaptic nerve terminal, VGCCs open in response to action potentials to allow an influx of calcium ions. The influx is a graded process, varying in a linear manner with the frequency of action potentials. An inhibition of the postsynaptic current was observed with L-type CCB (Ver) after electrical stimulation of dorsal nerve root [28]. Ver blocks the extracellular Ca^2+^ influx, reduces neurotransmitter release, decreases impulse conduction in nerve cells, and enhances the analgesic effect of EOZ. The results of the present study indicate that the onset and disappearance of toad sciatic nerve action potentially inhibited by EOZ and combined EOZ + Ver are fast and strong as compared to lidocaine. The mechanism may be the reason that effective components of EOZ are smaller molecules and more lipophilic chemical characteristics compared with lidocaine; these characteristics are conducive to molecules of EOZ penetrating effectively in nerve cells. The details of these mechanisms need further investigation.

## 5. Conclusion

Our result pertaining to the composition of essential oil has shown that linalool, limonene, and sabinene are the major components of essential oil from* Zanthoxylum schinifolium* Sieb. et Zucc. (EOZ). A synergistic interaction was observed between EOZ and verapamil in acetic acid-induced writhing test, hot plate test, tail flick test, and isolated toad sciatic nerve test. The results from this study can be extrapolated to clinical settings and additionally confirmed in different experimental models of pain, suggesting that this combination could be useful to treat diseases associated with pain in human beings. Further studies are necessary for the analgesic mechanism of EOZ and verapamil synergistic effect.

## Figures and Tables

**Table 1:** Main constituents of essential oil from *Zanthoxylum schinifolium* Sieb. et Zucc. determined by GS-MS.

Retention (min)	Compound name	Relative content (%)
7.356	.alpha.-Pinene	1.03
8.475	.beta.-Phellandrene	1.26
8.856	Sabinene	9.16
9.157	.beta.-Myrcene	3.87
9.835	.alpha.-Terpinene	1.05
10.158	Eucalyptol	1.64
10.262	d-Limonene	15.34
10.751	1,8-Cineole	1.05
11.122	.gamma.-Terpinene	1.02
12.671	Cis-sabinene hydrate	1.18
12.304	Linalool	32.54
13.154	Linalyl acetate	1.06
14.796	Terpineol	2.56
19.255	4-Terpinenol	1.78
22.038	.alpha.-Caryophyllene	1.41
22.847	Germacrene	1.32
23.469	.gamma.-Cadinene	1.03
	Different compounds	Trace (<1%)

**Table 2:** Effect of turning body induced by acetic acid in mice (*x* ± *s*, *n* = 10).

Group	Dosage (mg/kg)	潜 伏 期 latency of stomach ache/min	扭 体 次 数 amount of turning body/15 min
NS		2.8 ± 0.9	43.5 ± 13.8
EOZ low-dose	20 mg	3.0 ± 1.6③	40.7 ± 15.3③
EOZ middle-dose	40 mg	3.6 ± 1.8③	29.5 ± 5.6②
EOZ high-dose	80 mg	4.2 ± 1.7①	18.6 ± 6.4②
Ver	5 mg	3.9 ± 2.0③	19.6 ± 5.2②
EOZ + Ver low-dose	20 mg + 5 mg	4.3 ± 2.2①	19.2 ± 5.5②④
EOZ + Ver middle-dose	40 mg + 5 mg	4.5 ± 2.8①	18.3 ± 4.6②④
EOZ + Ver high-dose	80 mg + 5 mg	5.1 ± 3.1①	16.1 ± 4.8②④
Asp	200 mg	3.7 ± 1.3①	18.6 ± 5.5②

Values are presented as the mean ± S.E.M. (*n* = 10).

Compared with NS group: ①: *P* < 0.05, ②: *P* < 0.01, ③: *P* > 0.05; compared with relevant EOZ group: ④: *P* < 0.01.

**Table 3:** Analegsia effect of the pain induced by hot-plate in mice (*x* ± *s*, *n* = 10).

Group	Dosage (mg/kg)	Pain threshold of preadmin/s	Pain threshold of proadmin/s				
			15 min	30 min	60 min	90 min	120 min
NS		19.6 ± 5.8	19.3 ± 5.5	19.9 ± 5.3	20.3 ± 5.1	20.5 ± 5.5	19.8 ± 5.4
EOZ low-dose	20 mg	19.8 ± 4.7	20.5 ± 5.8③	20.9 ± 6.1③	20.8 ± 5.8③	20.8 ± 6.2③	20.5 ± 5.6③
EOZ middle-dose	40 mg	19.7 ± 4.8	23.9 ± 6.5③	31.4 ± 6.3①	32.2 ± 7.1①	34.8 ± 7.2①	34.6 ± 6.9①
EOZ high-dose	80 mg	19.7 ± 5.3	24.5 ± 5.6③	35.2 ± 5.3②	37.5 ± 6.1②	38.1 ± 5.6②	37.8 ± 5.3②
Ver	5 mg	20.3 ± 3.2	20.6 ± 5.2③	20.9 ± 6.1③	19.8 ± 6.4	21.5 ± 6.2③	21.6 ± 6.6③
EOZ + Ver low-dose	20 mg + 5 mg	20.5 ± 4.1	25.8 ± 6.6③	32.2 ± 5.7②④	33.5 ± 5.2②④	34.8 ± 5.9②④	35.5 ± 6.2②④
EOZ + Ver middle-dose	40 mg + 5 mg	19.6 ± 5.1	25.4 ± 5.2③	37.4 ± 5.3②④	40.6 ± 6.1②④	40.7 ± 6.1②④	41.5 ± 7.0②④
EOZ + Ver high-dose	80 mg + 5 mg	19.8 ± 4.8	26.2 ± 8.2③	41.2 ± 6.8②④	45.2 ± 6.8②④	46.7 ± 6.5②④	45.8 ± 7.2②④
Mor	10 mg	19.8 ± 4.6	45.3 ± 6.7②	46.8 ± 7.2②	45.3 ± 7.1②	24.9 ± 5.6③	21.7 ± 4.9③

Compared with NS group: ①: *P* < 0.05, ②: *P* < 0.01, ③: *P* > 0.05; compared with relevant EOZ group: ④: *P* < 0.01.

**Table 4:** Effect of the tail-curling latencies of mice in the warm water tail-flick test (*x* ± *s*, *n* = 10).

Group	Dosage (mg/kg)	Basal TFL/s	TFL of proadmin/s				
			15 min	30 min	60 min	90 min	120 min
NS		3.42 ± 0.52	3.48 ± 0.61	3.51 ± 0.72	3.62 ± 0.89	3.46 ± 0.61	3.48 ± 0.63
EOZ low-dose	20 mg	3.52 ± 0.68	3.72 ± 0.79③	4.16 ± 0.97③	4.25 ± 0.92③	4.20 ± 0.91③	4.25 ± 0.92③
EOZ middle-dose	40 mg	3.45 ± 0.62	4.15 ± 0.67③	4.85 ± 0.78①	5.01 ± 0.88①	5.12 ± 0.75①	5.12 ± 0.85①
EOZ high-dose	80 mg	3.50 ± 0.58	4.14 ± 0.85③	5.23 ± 0.82②	5.41 ± 0.76②	5.42 ± 0.83②	5.41 ± 0.86②
Ver	5 mg	3.49 ± 0.78	3.50 ± 0.75③	3.51 ± 0.82③	3.41 ± 0.84③	3.54 ± 8.6③	3.48 ± 0.85③
EOZ + Ver low-dose	20 mg + 5 mg	3.38 ± 0.72	4.56 ± 0.76③	6.85 ± 0.78②④	7.22 ± 0.75②④	7.34 ± 0.85②④	6.46 ± 0.78②④
EOZ + Ver middle-dose	40 mg + 5 mg	3.32 ± 0.65	4.12 ± 0.78③	9.23 ± 0.66②④	9.54 ± 0.78②④	9.68 ± 0.82②④	9.57 ± 0.65②④
EOZ + Ver high-dose	80 mg + 5 mg	3.47 ± 0.60	4.11 ± 0.73③	11.2 ± 0.78②④	12.4 ± 0.85②④	13.1 ± 0.87②④	12.5 ± 0.91②④
Mor	10 mg	3.56 ± 0.63	13.68 ± 0.86②	12.62 ± 0.76②	11.42 ± 0.79②	8.49 ± 0.88②	5.52 ± 0.82②

Compared with NS group: ①: *P* < 0.05, ②: *P* < 0.01, ③: *P* > 0.05; compared with EOZ group: ④: *P* < 0.05.

**Table 5:** Effect of amplitude of action potential (AP) of sciatic nerve in toads (*x* ± *s*, *n* = 5).

Group	Dosage (g/mL)	Preadmin/mV	Proadmin/mV			Time of AP vanishing
			1 min	5 min	10 min	min
RS		4.35 ± 1.25	4.26 ± 1.32	4.30 ± 1.16	4.33 ± 1.53	exist
EOZ	0.2%	4.39 ± 1.43	3.21 ± 0.85①③	0	0	4.98 ± 0.92③
Ver	0.05%	4.42 ± 1.02	4.39 ± 1.32	4.43 ± 1.06	4.29 ± 1.34	exist
EOZ + Ver	0.2% + 0.05%	4.45 ± 1.03	2.36 ± 1.02②③	0	0	2.62 ± 0.58③⑤
Lid	1%	4.53 ± 1.12	4.52 ± 1.35	4.50 ± 1.65①③	4.38 ± 1.65①③	22.89 ± 6.52③

Compared with preadministration: ①: *P* < 0.05, ②: *P* < 0.01; compared with NS group: ③: *P* < 0.05, ④: *P* < 0.01; compared with EOZ group: ⑤: *P* < 0.05.
